# Supplementary material for: The causal relationship between the human gut microbiota and pyogenic arthritis: a Mendelian randomization study
Source: Front Cell Infect Microbiol. 2024 Nov 26;14:1452480. doi: 10.3389/fcimb.2024.1452480 (PMC11629706; doi:10.3389/fcimb.2024.1452480)
Supplement: Supplementary file 1 [file DataSheet1.zip › mendelian test/ebi-a-GCST90027841.csv_forest.pdf]

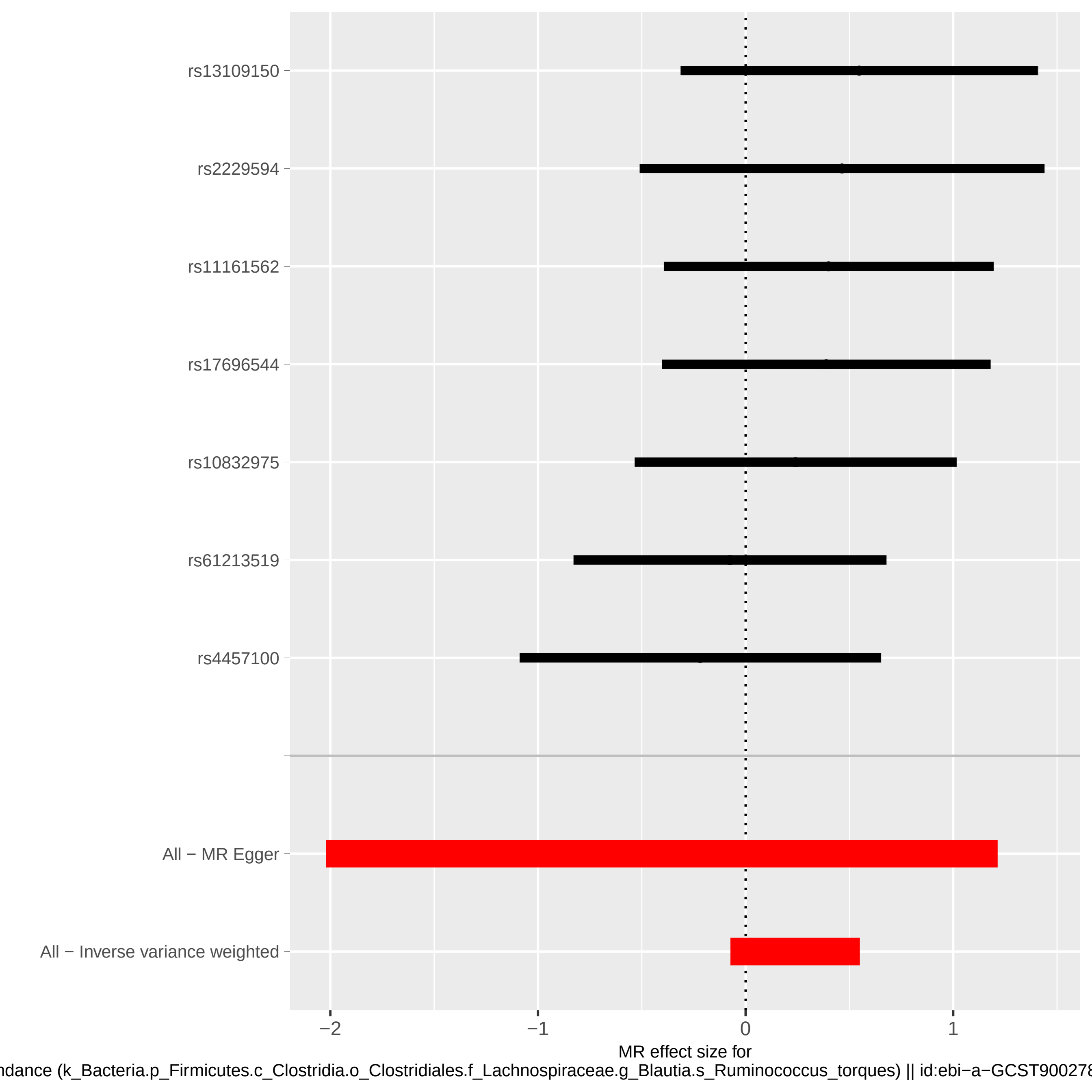

rs13109150
rs2229594
rs11161562
rs17696544
rs10832975
rs61213519
rs4457100
All − MR Egger
All − Inverse variance weighted
−2
−1
0
1
MR effect size for
ndance (k_Bacteria.p_Firmicutes.c_Clostridia.o_Clostridiales.f_Lachnospiraceae.g_Blautia.s_Ruminococcus_torques) || id:ebi−a−GCST90027
